# A generative adversarial network with multi-scale structural features for sparse-view photoacoustic tomography reconstruction

**DOI:** 10.1016/j.pacs.2026.100828

**Published:** 2026-04-28

**Authors:** Jialin Li, Xudong Luo, Yiming Ma, Gang Li, Tingting Li, Zheng Yuan, Yi Shen, Cheng Ma, Mingjian Sun

**Affiliations:** a Department of Control Science and Engineering, Harbin Institute of Technology, Harbin, Heilongjiang 150001, China; b Department of Control Science and Engineering, Harbin Institute of Technology, Weihai, Shandong 264200, China; c Harbin Institute of Technology Suzhou Research Institute, Suzhou, Jiangsu 215000, China; d Harbin Institute of Technology (Weihai) Qingdao Innovation and Development Base, Qingdao, Shandong 266109, China; e Shandong Laboratory of Advanced Biomaterials and Medical Devices in Weihai, Weihai, Shandong 264209, China; f Department of Electronic Engineering, Tsinghua University, Beijing 100084, China

**Keywords:** Photoacoustic tomography, Sparse-view imaging, Generative adversarial network

## Abstract

Photoacoustic tomography combines high optical absorption contrast with deep ultrasonic penetration, providing powerful capabilities for high resolution biomedical imaging. It often demands wide field and large data acquisition, posing significant challenges to imaging speed. Sparse sampling effectively reduces data volume and accelerates imaging, but the resulting artifacts and detail loss limit its applications. Therefore, we construct a sparse-view photoacoustic tomography system and propose generative adversarial network with multiscale structural features, to achieve high speed and quality three-dimensional imaging. The generator incorporates pyramid squeeze attention block to extract multiscale structural information, while skip connection with a channel attention module in the discriminator enhances organ structural consistency. In addition, dual gradient regularized adversarial loss is designed to improve stability in detail enhancement and artifact suppression. Experiments with 128 and 64 views sampling verify that the proposed system achieves superior artifact suppression and detail recovery, preserving structural features consistent with full-view reconstruction. Furthermore, compared with the strongest baseline diffusion model and MambaIR, MSF-GAN improved the peak signal-to-noise ratio and structural similarity index by 1.128% and 1.176% under 128 views sampling, and by 2.442% and 0.536% under 64 views. These demonstrate that the proposed system and method effectively leverage photoacoustic structural information to improve fidelity and contrast. This work achieves a desirable balance between high speed and quality reconstruction, providing a promising pathway for translating photoacoustic tomography into practical clinical applications

## 1 Introduction

Photoacoustic tomography (PAT) is a non-invasive imaging technique that combines optical absorption contrast with the deep penetration capability of ultrasound, enabling high contrast visualization of deep tissues over wide field view. It is particularly suitable for *in vivo* animal imaging, showing unique advantages in biomedical applications such as tumor and brain functional imaging [1], [2], [3]. In recent years, significant advances have been achieved in PAT, including improvements in imaging system design, reconstruction algorithms, and functional imaging, further enhancing its performance and expanding application scope [4], [5]. PAT systems typically employ circular pulsed laser illumination and circular-array transducers for acoustic detection, and have evolved from two-dimensional to three-dimensional imaging, leading to a rapid increase in acquisition channels and data volume [6], [7], [8]. It demands on real time performance and data storage. Sparse sampling has become a key technique to balance imaging speed and quality. However, sparse sampling often leads to artifacts and detail loss during reconstruction, which severely limits its application in practical systems [9], [10]. Therefore, it is importance to achieve efficient PAT reconstruction under sparse-view conditions.

Currently, strategies for suppressing artifacts in sparse-view PAT reconstruction can be divided into two categories: traditional reconstruction and deep learning-based methods. Traditional reconstruction techniques such as filtered back-projection (FBP) and time reversal (TR) are easy to implement, but they tend to produce directional streak artifacts and aliasing under sparse sampling [11], [12]. Iterative methods based on physical models suppress sparse reconstruction artifacts by introducing regularization terms, such as Tikhonov, total variation (TV), and other regularized models [13], [14], [15]. Although these approaches have strong physical consistency, their performance heavily depends on prior information and parameter tuning. They also lead to slow computation and excessive smoothing of complex structures, which limits their applicability in high speed PAT imaging. Deep learning-based reconstruction methods have recently emerged as promising direction. Convolutional neural networks (CNNs) can learn local structural priors from data to restore image information, but their limited receptive fields may lead to over smoothing and stripe artifacts [16]. The U-Net architecture improves detail recovery through multiscale feature fusion and local convolutions, but they may still lead to image quality decrease [17]. Transformer models introduce self-attention to capture long range dependencies and global context, but they often face high computational demands and a tendency to overfit [18]. Recently, diffusion models have been used in medical image reconstruction due to their powerful generative priors [19]. Song et al. combined a score-based diffusion model with model-based iteration to improve sparse-view PAT reconstruction [20], while Li et al. introduced a sinogram-domain prior guided enhanced diffusion framework for ultra sparse PAT [21]. But their high inference latency increases the overall complexity of the reconstruction process. In PAT sparse reconstruction, deep learning approaches have shown advantages in straightforward deployment and fast inference. But they remain dependent on the quality and distribution of training data. Under extreme sparsity, they often generate spurious details, which restricts their effectiveness in *in vivo* PAT imaging.

In recent years, generative adversarial networks (GANs) have been widely applied in biomedical imaging, exhibiting strong perceptual quality and structural fidelity. Their core principle is adversarial learning between generator and discriminator, where the generator reconstructs structural and textural information, while the discriminator evaluates differences at global and local scales, thereby enhancing structural consistency [22], [23]. This allows GANs to capture more realistic image details under complex background conditions, achieving image restoration and artifact suppression, outperforming CNNs and U-Nets. In addition, compared with diffusion models, GANs provide a more favorable trade-off between computational efficiency and structural fidelity. Owing to these advantages, GANs have been applied to various medical imaging modalities such as computed tomography, magnetic resonance imaging, ultrasound imaging, and optical microscopy [24], [25], [26], [27]. For PAT, Huang et al. proposed FDP-GAN to improve sparse view reconstruction by integrating multisource information, thereby enhancing image fidelity and suppressing artifacts [28]. Zhang et al. proposed FD-UGAN, which enhances sparsely sampled photoacoustic reconstruction with redesigned encoding and decoding pathways for multiscale feature extraction and integration [29]. Dong et al. also designed an attention driven conditional GAN for PAT image restoration under blur and streak artifacts [30]. Therefore, applying GANs to sparse-view PAT reconstruction offers significant potential. The key challenge is how to design a sparse-view reconstruction strategy that fully leverages structural information in PAT images, achieving high quality reconstruction results comparable to full-view acquisition.

In this work, we develop a sparse-view photoacoustic tomography system and propose a generative adversarial network with multiscale structural feature (MSF-GAN) for high resolution sparse-view reconstruction. First, we establish a PAT system specifically designed for sparse-view acquisition, while still maintaining high quality *in vivo* animal imaging. Second, a pyramid squeeze attention block is incorporated into the generator, and a skip connection with channel attention module is integrated into the discriminator, to improve the accuracy and consistency of organ structural contour recovery. Third, a dual-gradient regularized adversarial loss is designed to enhance detail restoration and artifact suppression. To validate the performance of the proposed system and method, a series of simulation and *in vivo* experiments were conducted under various sparse-view conditions. Experimental results show that MSF-GAN recovers structural texture and boundary features consistent with full-view imaging, significantly improving the peak signal-to-noise ratio (PSNR) and structural similarity index (SSIM) under sparse sampling. This study provides a framework for high speed and precision *in vivo* animal imaging and offers broad prospects for biomedical applications.

## 2 Methods

This section presents the sparse-view reconstruction method for PAT, along with the experimental system and configurations used in this study. First, we describe the principle and core formulation of MSF-GAN. Then, we present the sparse-view PAT system, including hardware modules and system parameters. Finally, we introduce the configurations of the simulation and *in vivo* experiments.

### 2.1 Sparse-view photoacoustic tomography reconstruction model

**Fig. 1:**
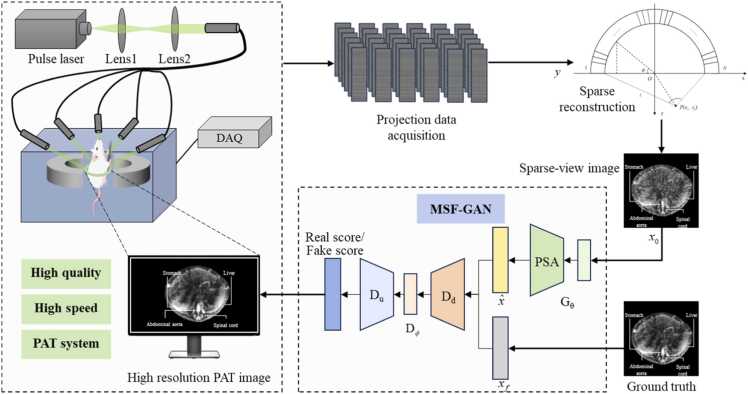
Schematic diagram of sparse-view photoacoustic tomography reconstruction.

To achieve high quality reconstruction under sparse sampling conditions, we develop a sparse-view photoacoustic tomography system and propose a generative adversarial network [23] incorporating multi-scale structural features. The overall framework of sparse-view PAT reconstruction is illustrated in Fig. 1. Frist, we acquire sparse-view photoacoustic projections y using the developed PAT system. Specifically, the pulsed laser induces optical absorption and thermoelastic expansion in the tissue, generating an initial pressure distribution $p_{0}(r)$:

(1) $$p_{0}(r)=\Gamma \mu_{a}(r)\Phi (r),$$

where Γ is Grüneisen parameter, $\mu_{a}(r)$ is optical absorption coefficient, and Φ(r) is the optical fluence. After discretization, the sparse-view projections from the detectors are written as a matrix $y\in {\mathbb{R}}^{M}$. The PAT forward model can be expressed as:

(2) y=Ax+n,

where A and n are the system matrix and noise. Then, the sparse-view projections y are reconstructed into initial images $x_{0}$ using delay-and-sum (DAS) beamforming [31],

(3) $$x_{0}=R_{D}(y),$$

where $R_{D}(\cdot )$ denotes the DAS reconstruction. The sparse-view PAT only measures projections from a subset of detector positions, which leads to artifacts when using DAS reconstruction. To recover structural details and suppress artifacts, the initial PAT image $x_{0}$ serves as the input to the network. Then, MSF-GAN enhances the precision of sparse-view reconstruction, by integrating the enhanced generator, discriminator, and loss function.

### 2.2 Generator network architecture

**Fig. 2:**
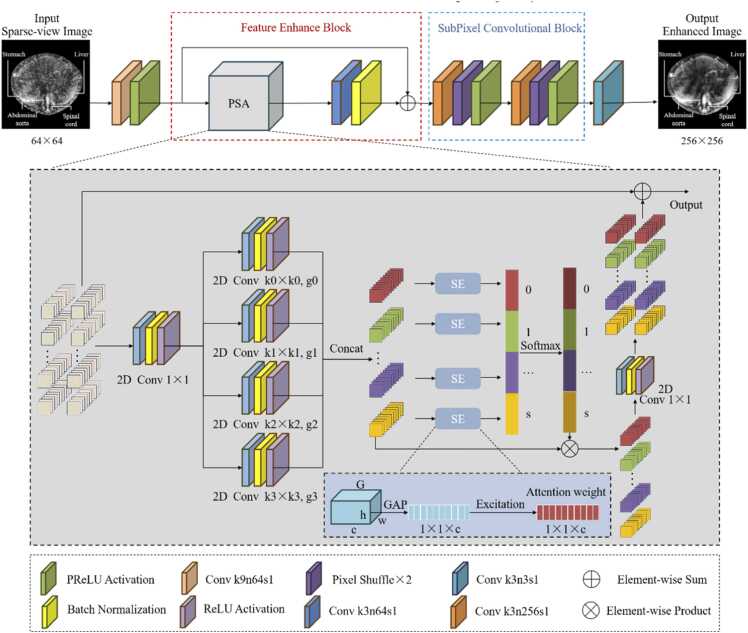
The architecture of the proposed generator.

To enhance detail recovery and artifact correction in sparse photoacoustic reconstruction, we redesign the traditional generator structure by introducing a pyramid squeeze attention (PSA) module [32]. The architecture of generator $G_{\theta }$ is shown in Fig. 2. PSA can acquire richer photoacoustic structural feature representation and learn global dependencies across feature maps. It is particularly suitable for capturing and restoring complex high frequency details in sparse-view photoacoustic reconstruction.

First, the sparse-view photoacoustic image $x_{0}$ is processed through convolution and PReLU activation [33] for shallow feature encoding:

(4) $$F_{0}=\varphi (W_{s}*x_{0}+b_{s})$$

where $W_{s}$ and $b_{s}$ are the weights and bias, * denotes convolution, and φ(⋅)denotes the PReLU activation function. Then, the feature map $F_{0}\in {\mathbb{R}}^{H\times W\times C}$ passes through a feature enhance block consisting of PSA, convolution, and batch normalization (BN) layers to extract structural features $F_{r}$. To stabilize training and preserve low frequency information, a residual fusion is applied to obtain consistent feature $F_{e}=F_{0}+F_{r}$.

In PSA module, the feature map $F_{0}$ is first processed using 1× 1 convolutions followed by BN and ReLU activation [34] to perform channel remapping. Four parallel convolutional branches with different kernel sizes are applied, where each scale produces an intermediate feature map $F_{s}\in {\mathbb{R}}^{H\times W\times C_{b}}$:

(5) $$F_{s}={\mathrm{Conv}}_{k_{s}\times k_{s}}^{\left(s\right)}(\phi ({\mathrm{BN}}_{0}({\mathrm{Conv}}_{1\times 1}^{\left(0\right)}(F_{0})))),$$

where ${\mathrm{Conv}}_{k_{s}\times k_{s}}^{\left(s\right)}(\cdot )$ denotes convolution with kernel size $k_{s}\times k_{s}$, ϕ(⋅) is the ReLU activation function. These maps cover distinct receptive fields ranging from fine textures to global structures. Then, each branch is followed by a squeeze and excitation mechanism (SE) model to calculate channel weights. In SE module, global average pooling is used to squeeze the spatial dimensions of $F_{s}$ into a channel descriptor $z_{s}\in {\mathbb{R}}^{C_{b}}$. And a fully connected gating mechanism generates the channel attention vector $a_{s}\in {\mathbb{R}}^{C_{b}}$:

(6) $$a_{s}=\sigma (W_{2}^{\left(s\right)}\delta (W_{1}^{\left(s\right)}z_{s})),z_{s,c}=\frac{1}{HW}\sum_{i=1}^{H}\sum_{j=1}^{W}{\left[F_{s}\right]}_{i,j,c},$$

where $W_{1}^{\left(s\right)}$ and $W_{2}^{\left(s\right)}$ represent weight matrices, δ and σ are activation functions. The SE responses are further normalized along the scale dimension by a Softmax operation for each channel, and the reweighted multiscale features $\hat{F}$ are obtained by:

(7) $${\hat{F}}_{s}(i,j,c)=F_{s}(i,j,c)\cdot \frac{\exp(a_{s,c})}{\sum_{t=0}^{3}\exp(a_{t,c})}.$$

The $\hat{F}$ from all branches are stacked along the channel dimension and transformed to the original channel number by a 1×1 convolution followed by BN and ReLU activation:

(8) Fp=F0+ϕ(BN1(Conv1×1(1)(Concat(F^0,F^1,F^2,F^3)))).

In this way, the proposed PSA block allows the generator to adaptively adjust feature map weights according to multiscale information. The resulting features are then refined by a convolution and BN enhancement block, and combined with a residual connection, generating the enhanced feature *F*_*e*_ for the following reconstruction. Notably, as the PSA module performs channel reweighting after parallel convolutions with different receptive fields, directly transforming the input features may lead to over modulation of structural responses. The internal residual connection introduced in the PSA module can stabilize the learning of multiscale attention. Meanwhile, considering the low frequency structural components in photoacoustic images, the network should remain focused on the correction of sparse-view artifacts. The external residual connection in the feature enhancement block can preserve stable base features while optimizing the overall structural representations.

Subsequently, the feature $F_{e}$ is up sampled by a subpixel convolutional block consisting of two cascaded units, each consisting of convolutional layer, pixel shuffle operation, and PReLU activation. This design enables up sampling from the compact feature space to the image space, effectively reducing checkerboard artifacts and enhancing the recovery of fine photoacoustic structures. Finally, a 3 × 3 convolution maps the features to the target channel, to produce an intermediate enhanced sparse-view reconstruction image $\hat{x}$. The proposed generator integrates feature enhancement block and convolutional block, fully exploits multiscale structural information in photoacoustic images. Together with the improved discriminator and loss function, it can preserve structural details and suppress artifacts under sparse-view PAT reconstruction.

### 2.3 Discriminator network architecture

**Fig. 3:**
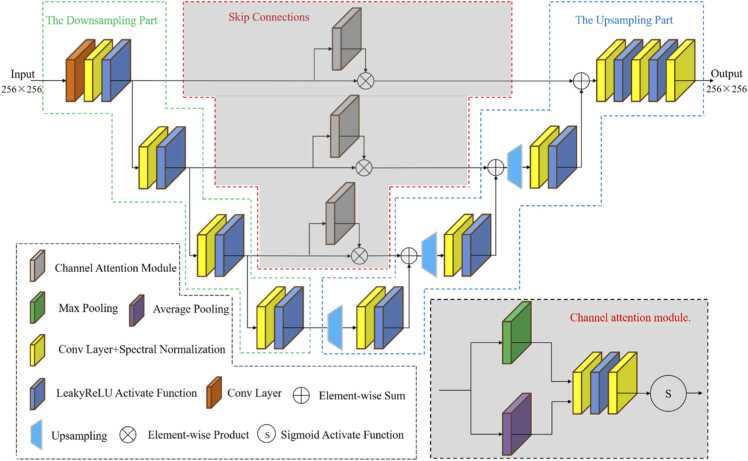
The architecture of the proposed discriminator.

To improve detail discrimination in biological structures, the discriminator in the GAN framework is also redesigned. The architecture of discriminator is shown in Fig. 3. Given an input PAT image, the structure-aware discriminator $D_{\phi }(x)$ outputs a spatially resolved authenticity map S, enabling the network to evaluate structural fidelity. It consists of three main components: down sampling encoder, skip connection module, and up sampling decoder. The encoder extracts hierarchical sparse reconstruction features, while the decoder gradually restores spatial resolution and refines high frequency details. Skip connection module is combined with channel attention module (CAM) [35] to enhance feedback precision. It enables the discriminator to provide both pixel and global level feedback for more accurate learning signals, improving the consistency of sparse-view PAT reconstruction.

The encoder of the discriminator consists of cascaded modules of convolution, spectral normalization, and LeakyReLU activation [36]. The first convolution uses stride two to expand the receptive field rapidly. Spectral normalization and LeakyReLU activation improve training stability and fitting capability. This stage extracts sparse reconstruction artifact features while preserving contrast and morphological information, improving global modeling ability. The decoder focuses on stabilizing the evaluation of local authenticity, producing consistent tissue boundaries and textures. It performs bilinear up sampling to enlarge feature maps and restore spatial resolution step by step, followed by convolution and activation operation to extract high frequency structural information. The final convolution layer transforms the decoder features into a realness map, enhancing structures discrimination and suppresses artifact interference.

Based on the U-Net architecture in discriminator [37], the skip connection with channel attention module suppresses redundant interference introduced by down sampling. Specifically, it is achieved by inserting CAM into each skip connection layer to improve sensitivity to key tissue regions within *in vivo* animals. The CAM learns importance weights across feature map channels, directing the discriminator to focus on regions that contain true structural textures. For the feature map $F_{e}^{\left(l\right)}$ of the *l*-th channel, the CAM first aggregates global spatial information by average and max pooling:

(9) $${f_{a\mathrm{vg}}}^{\left(l\right)}=AP(F_{e}^{\left(l\right)}),{f_{\max}}^{\left(l\right)}=MP(F_{e}^{\left(l\right)}),$$

where AP(⋅)and MP(⋅) denote average pooling and max pooling, respectively. The two channel feature vectors are passed through a shared two-layer network to generate the channel attention weights. Specifically, the channel attention map $M_{c}^{\left(l\right)}$is computed as:

(10) $$M_{c}^{\left(l\right)}=\delta (W_{2}^{\left(l\right)}L(W_{1}^{\left(l\right)}{f_{avg}}^{\left(l\right)})+W_{2}^{\left(l\right)}L(W_{1}^{\left(l\right)}{f_{\max}}^{\left(l\right)})),$$

where $W_{1}^{\left(l\right)}$and $W_{2}^{\left(l\right)}$ are weight matrices, δ(⋅)is Sigmoid activation function, and L(⋅) represents convolution, spectral normalization and LeakyReLU activation function. Then, the original encoder feature map is rescaled, and the refined skip feature is combined with the decoder feature. The skip fusion is given by:

(11) $${{\hat{F}}_{e}}^{\left(l\right)}=M_{c}^{\left(l\right)}⊙F_{e}^{\left(l\right)},F_{d}^{\left(l\right)}=U(F_{d}^{\left(l+1\right)})+{{\hat{F}}_{e}}^{\left(l\right)}$$

where ⊙ denotes element-wise multiplication, and U(⋅)consists of bilinear up sampling followed by convolution, spectral normalization and LeakyReLU activation, which recovers spatial resolution while refining local structures.

The CAM mechanism enables the discriminator to provide both pixel and global level feedback across different regions, emphasizing discriminative channels relevant to related to fine and overall structures. By integrating CAM into skip connections, the proposed discriminator validates structural authenticity and global consistency more accurately, providing adversarial feedback and preserving fine structures in sparse-view PAT reconstruction.

### 2.4 Loss function

During training, the generator and discriminator engage in adversarial learning based on a relativistic pairing loss function, which improves stability and reconstruction quality in sparse imaging. Traditional GAN loss functions may fail to learn accurate structural details when faced with blurred regions [38]. To address this, the proposed loss function incorporates two gradient regularization terms to control the response strength of the discriminator.

To better compare the relative realism between full-view reconstruction images $x_{f}$and generated images$\hat{x}$, the relativistic adversarial loss can be defined as:

(12) $$L_{\mathrm{Rp}}(\theta ,\phi )=E_{\left(x_{0},x_{f}\right)}\left[-\;\log\;\sigma (D_{\phi }\left(x_{f}\right)-D_{\phi }\left(\hat{x}\right))\right],\;\hat{x}=G_{\theta }\left(x_{0}\right),$$

where $x_{0}$ and$x_{0}$ denote the generator and discriminator respectively, $x_{0}$ is sparse-view reconstruction image, σ(⋅) is the Sigmoid function and $E_{\left(x_{0},x_{f}\right)}\left[\cdot \right]$ denotes the expectation. It focuses on the perceptible differences between full-view image and generated image, increasing sensitivity to regions with structural variations. Therefore, $L_{\mathrm{Rp}}$ can effectively guide the generator to perform targeted corrections in high frequency structures such as boundaries and tissue.

To prevent gradient explosion and overfitting in the discriminator, especially under sparse-view conditions, two gradient regularization terms are introduced:

(13) $$R_{1}\left(\phi \right)=\frac{\gamma_{1}}{2}E_{x_{f}}\left[{\left\|\nabla_{x_{f}}D_{\phi }\left(x_{f}\right)\right\|}_{2}^{2}\right],$$

(14) $$R_{2}(\theta ,\phi )=\frac{\gamma_{2}}{2}E_{x_{0}}\left[{\left\|\nabla_{\hat{x}}D_{\phi }\left(\hat{x}\right)\right\|}_{2}^{2}\right],\hat{x}=G_{\theta }\left(x_{0}\right),$$

where ∇ and ∇ are hyperparameters controlling the weighting of two regularization terms, ∇ denotes the spatial gradient. *R*_1_ constrains the gradient response of the discriminator on full-view reconstruction images $x_{f}$, preventing overfitting to the data distribution. *R*_2_ constrains the unstable local gradient responses on generated images $\hat{x}$, improving the convergence of the adversarial training process. The final total loss function is expressed as:

(15) $$L_{\mathrm{total}}(\theta ,\phi )=L_{\mathrm{Rp}}(\theta ,\phi )+\lambda_{1}R_{1}(\phi )+\lambda_{2}R_{2}(\theta ,\phi ),$$

where$\beta_{1}=0.9$ and$\beta_{1}=0.9$ are the weighting coefficients for regularization terms, balancing training stability and reconstruction quality. This loss function integrates the structure aware mechanisms of both generator and discriminator, improving the stability of the framework in detail enhancement and artifact suppression. In summary, the proposed MSF-GAN combines optimized generator, feature sensitive discriminator, and loss function with dual gradient regularization. It effectively utilizes structural information, ensuring accurate recovery of boundaries and texture details in sparse-view PAT reconstruction.

### 2.5 Sparse-view photoacoustic tomography system

**Fig. 4:**
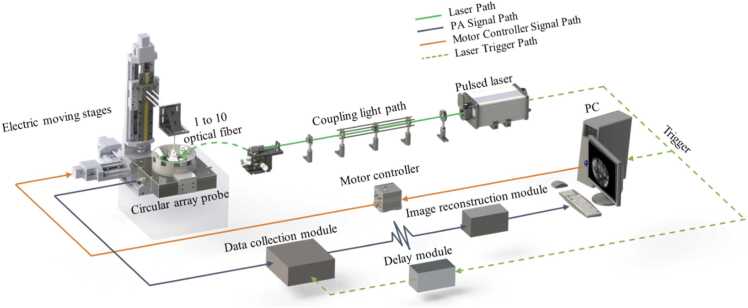
Schematic diagram of the sparse-view photoacoustic tomography system.

The sparse-view photoacoustic tomography system is illustrated in Fig. 4. The system comprises four key components: laser excitation module, acoustic detection module, data acquisition module, and reconstruction module. A nanosecond pulsed optical parametric oscillator laser (SpitLight 600, Innolas, Germany) is used as the excitation source. It provides a tunable wavelength range of 680–980 nm, enabling multi-wavelength and contrast imaging. The laser beam is first collimated and shaped, then evenly distributes onto the sample surface via a 1-to-10 optical fiber, achieving uniform circular illumination. The imaging region is concentric with the circular transducers, to ensure spatial alignment between optical excitation and acoustic detection. The photoacoustic signal is detected by two 128-element circular transducer arrays, and acquired using an ultrasound imaging platform (Prodigy, S-Sharp, China). The raw signals are digitally filtered and then passed to the reconstruction module, ultimately reconstructing photoacoustic tomography images.

**Fig. 5:**
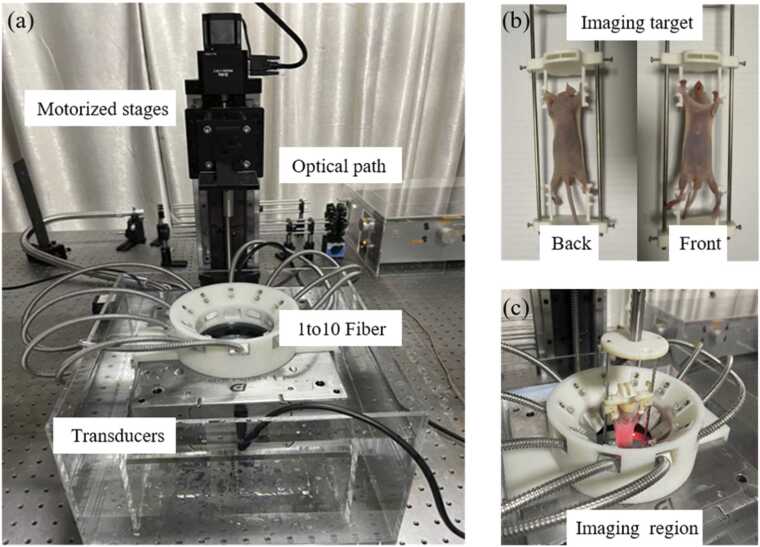
Circular-array photoacoustic tomography system: (a) PAT system;(b) imaging target;(c) imaging region.

The configuration of imaging region plays a decisive role in imaging quality, as shown in Fig. 5. The system consists of two semicircular transducer arrays with a central circular region of 55 mm in diameter, sufficient to accommodate a small animal or standard phantom target. The ultrasound transducers are composed of two 128 channel arrays of piezoelectric elements with 80% bandwidths. Their center frequencies are 5.5 MHz and 2.5 MHz respectively, which provide a complementary acoustic detection strategy for *in vivo* photoacoustic tomography. Specifically, the 5.5 MHz array provides a higher axial resolution, with a theoretical value of approximately 175 μm, although its effective imaging depth is usually limited to about 20 mm. It is more sensitive to high frequency components, which helps recover fine superficial tissue details. In contrast, the 2.5 MHz array has a lower theoretical axial resolution of approximately 385 μm, but its imaging depth can reach about 50 mm under different tissue conditions. It can detect signals from deeper structures, enabling effective detection of deep organ regions. This multi-band frequency configuration improves the adaptability of the system to complex biological tissues [39]. The configuration ensures high spatial resolution and broad detection coverage. When the imaging targe is positioned in the imaging region, laser excitation and acoustic detection are synchronized temporally and spatially. The timing and synchronization module precisely synchronizes laser emission, signal acquisition, and stage motion, ensuring temporal coordination for three-dimensional photoacoustic data reconstruction.

**Fig. 6:**
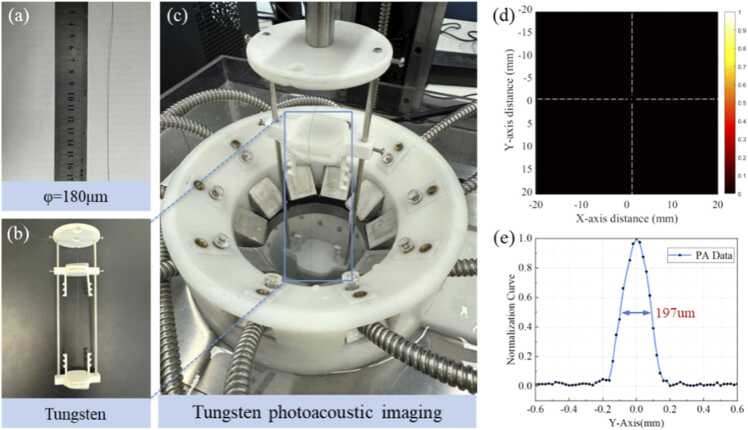
The resolution of the photoacoustic tomography system: (a)-(c) tungsten and PAT imaging; (d) tungsten reconstruction image; (e) the normalization curve corresponding to the horizontal line in (d).

To evaluate the resolution of the PAT system, a tungsten wire with a diameter of 180μm was employed as the standard target. Figs. 6(a)-6(c) illustrate the diameter of the tungsten and its measurement procedure. The tungsten was vertically positioned at the center of the imaging region. Fig. 6(d) presents the reconstructed tungsten wire image for extracting intensity profile. The full width at half maximum (FWHM) of the photoacoustic signal profile was calculated to quantify the imaging resolution. Fig. 6(e) shows the FWHM of the signal intensity corresponding to the horizontal dashed line in Fig. 6(d). The measured resolution of the system was 197μm, demonstrating PAT system can meet the requirements for high resolution imaging of tissue structures.

### 2.6 Sample preparation and arrangement

To validate the effectiveness of the proposed sparse reconstruction method, preliminary numerical simulations were conducted. The simulations were implemented using the k-Wave toolbox developed by Treeby [40], which accurately modeled the propagation and projection of photoacoustic signals. A vascular structure was used as the simulation model, with the initial acoustic pressure source defined by the simulated vascular configuration. The computational domain was set to 256 × 256 grid points. A circular array with a 4.5 mm radius and 256 uniformly distributed elements was used to form a complete 360° detection angle. The central frequencies of the transducer elements were 5.5 MHz and 2.5 MHz with 80% bandwidth. The propagation medium was assumed to be water, with a sound speed of 1500 m/s and density of 1000 kg/m³ . The configuration effectively simulates the propagation of photoacoustic signals generated by vascular structures, providing a foundation for evaluating the reconstruction algorithm.

To further demonstrate the effectiveness of MSF-GAN and its applicability in the developed PAT system, *in vivo* photoacoustic tomography experiments were conducted on animals. BALB/c Nude mice (8–12 weeks, 20 g, Charles River Laboratories) were selected as the animal model. To minimize interference, each mouse was fasted for 12 h before imaging, with water access maintained to clear intestinal contents. Body hair was removed to reduce epidermal scattering signals. During the experiment, 800 nm laser was employed for photoacoustic signal excitation. The laser fluence was satisfied ANSI biological safety standards. The mouse was anesthetized using an isoflurane anesthesia system (RWD R500IP, China) with air flow rate of 1 L/min and isoflurane concentration of 0.75%. Each mouse was then fixed on a custom holder for imaging. The animal body was immersed in 36°C water bath to ensure efficient acoustic coupling and prevent hypothermia induced stress. All experiments were performed according to protocols approved by Laboratory Animal Care Committee.

### 2.7 Implementation details

The datasets include both simulation dataset and *in vivo* dataset. For the simulation dataset, we used the k-Wave toolbox to generate a total of 500 vascular models with multiscale structural variations. For each initial pressure distribution, we simulated sparse-view acoustic signals using 128-element and 64-element arrays, and reconstructed ground truth images using the DAS method. The sparse-view images were paired with their corresponding binary images. The simulation dataset was randomly divided into training, validation, and testing sets with a ratio of 8:1:1. To ensure MSF-GAN was applicable to the sparse-view PAT system and maintain stability during training, the *in vivo* dataset was constructed using the system. The dataset consisted of 500 mouse images under full-view acquisition. Since adjacent slices from the same mouse are highly correlated, the dataset was split at the mouse level to avoid potential data leakage. Specifically, images from eight mice were used for training and one mouse for testing, corresponding to 400 and 50 images. In addition, images from another mouse were used as a validation set for model selection during training. To increase structural diversity without altering intrinsic characteristics of photoacoustic data, geometric augmentations such as random rotation and flipping were applied to the training set, increasing its size to four times. The testing set was used for quantitative comparisons under different sparse-view conditions.

The proposed model was implemented in PyTorch and trained on a workstation equipped with AMD Ryzen 7 4800 H CPU, 16 GB RAM, and NVIDIA GeForce GTX 1650 GPU. Based on experimental evaluations, in the loss function, the hyperparameters of gradient regularization term $\beta_{1}=0.9$ and $\beta_{1}=0.9$were set to 0.5 and 1, while weight coefficients $\beta_{1}=0.9$ and $\beta_{1}=0.9$were set to 1 and 0.8. The batch size was set to 8. We used Adam optimizer with $\beta_{1}=0.9$ for optimization and alternately update the generator and discriminator network. The network was trained for 200 epochs, and the model with the best validation performance during training was saved for subsequent evaluation. The initial learning rate was set to $1\times 10^{-4}$ for 150 epochs, and was halved.

To evaluate the performance, the proposed method was compared with several classical reconstruction algorithms, including TV, U-Net, Swin Transformer [41], as well as photoacoustic sparse reconstruction methods such as WGAN-GP [42] PA-GAN [43], MambaIR [44] and diffusion model [45]. The raw projection data of the *in vivo* dataset were first directly reconstructed to obtain baseline imaging results, as shown in supplementary Fig. S1, which reflect the native imaging performance of the developed system under sparse-view conditions. To facilitate subsequent model training, post-processing operations were applied to the *in vivo* dataset to preliminarily improve dataset quality. Specifically, all raw projection data were preprocessed using bandpass filtering to suppress noise interference before being fed into the reconstruction module. During reconstruction, a homogeneous acoustic model was assumed and the speed of sound was set to 1500 m/s, which is a commonly used approximation for soft biological tissues in photoacoustic imaging. After reconstruction, the images were further processed using the Hilbert transform and automatic edge segmentation. The processed results are presented in supplementary Fig. S2, demonstrating the improvement in image quality and structural visibility. For TV method, the regularization parameter λ was determined separately for different sparse-view sampling settings. Considering the balance between reconstruction quality and computational cost, λ was set to 0.08 for 128 views reconstruction and 0.1 for 64 views reconstruction. And the same value was applied to all images within the corresponding setting. All comparative methods were reproduced and trained under their original configurations to ensure fairness in comparison.

## 3 Experiments and results

### 3.1 Sparse-view photoacoustic tomography reconstruction based on simulated vessels

**Fig. 7:**
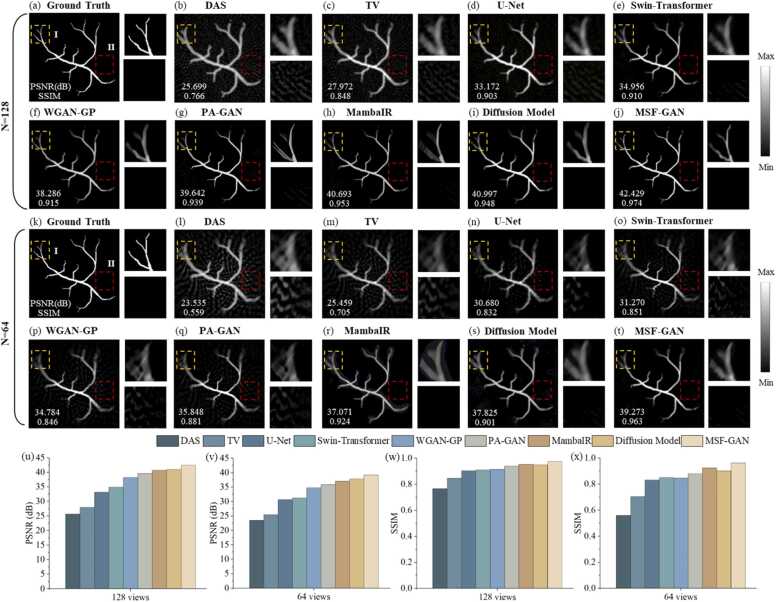
Reconstruction results of simulated blood vessels: (a) ground truth; (b)-(j) reconstruction results of DAS, TV, U-Net, Swin-Transformer, WGAN-GP, PA-GAN, MambaIR, Diffusion Model and MSF-GAN under 128 views; (k) ground truth; (l)-(t) reconstruction results of DAS, TV, U-Net, Swin-Transformer, WGAN-GP, PA-GAN, MambaIR, Diffusion Model and MSF-GAN under 64 views; (u)-(x) PSNR and SSIM of reconstruction images under 128 and 64 views.

To evaluate the performance of the proposed MSF-GAN in sparse-view photoacoustic tomography reconstruction, we built simulated blood vessels and conducted experiments and quantitative analysis under 128 and 64 views. Figs. 7(a) and 7(k) were the ground truth. Figs. 7(b)-7(j) showed the sparse-view reconstructions and the corresponding regions of interest under 128 views for DAS, TV, U-Net, Swin-Transformer, WGAN-GP, PA-GAN, MambaIR, diffusion model and MSF-GAN. Figs. 7(l)-7(t) showed the reconstruction results and magnified regions under 64 views. Comparing the results under the two sparse views reflected differences among methods in artifact suppression and structure preservation.

Overall, sparse sampling introduced artifacts in PAT reconstruction. The artifacts became stronger as the number of views decreased and may even mask vessel signals in Figs. 7(b) and 7(l). The TV method suppressed part of the artifacts, but performed poorly at 64 views in Fig. 7(m). In regions of interest II, background artifacts remained obvious because TV had difficulty distinguishing thin true vessels from stripe artifacts. U-Net and Swin-Transformer increased the overall PSNR at different view settings, but showed over smoothing. In regions of interest I in Figs. 7(n) and 7(o), vessel edges became blunt and textures were blurred at 64 views. WGAN-GP and PA-GAN enhanced edges and textures were close to the ground truth at 128 views in Figs. 7(f) and 7(g). However, as shown in magnified regions I, there were excessive enhancement of distal vessel and artifact blending. Under extremely sparse sampling, background artifacts still appeared in the gaps between vessels and background in regions II, likely due to overfitting and insufficient structural constraints. In addition, MambaIR and the diffusion model showed good background noise suppression under both 128 views and 64 views. As shown in Figs. 7(h), 7(i), 7(r), and 7(s), both methods effectively reduced the background interference in region II. However, a certain degree of overfitting was still observed in the vascular region of region I, where the fine structures deviated from the true morphology. This issue became more obvious under 64 views. In contrast, MSF-GAN achieved the best reconstruction at both 128 and 64 views. The vessel network was continuous and clear. The magnified regions I and II showed that MSF-GAN effectively removed artifacts, while accurately restoring complex vascular structures such as bifurcations and edges in Figs. 7(j) and 7(t). These results indicated that MSF-GAN preserved textures and edge features consistent with full-view reconstruction.

To further quantify the sparse reconstruction performance of MSF-GAN, Figs. 7(u)-7(x) presented PSNR and SSIM across different methods under 128 and 64 views. PSNR measures the pixel error between the reconstruction and the reference. SSIM reflects structural similarity and perceptual quality. Quantitative results showed that under 128 views, MSF-GAN achieved the highest scores among all methods. PSNR and SSIM values reached 42.429 dB and 0.974, improving over the sparse input baseline by about 65% and 27%. Even under 64 views, MSF-GAN still achieved PSNR of 39.273 dB and SSIM of 0.963. It indicated that MSF-GAN maintained stable reconstruction performance across different sparsity levels. Quantitative and qualitative results demonstrated that MSF-GAN effectively exploited multiscale structural information in photoacoustic images, achieving artifact removal and detail enhancement. The method improved image quality for sparse-view PAT reconstruction, offering reliable technical support for high speed imaging.

### 3.2 Sparse-view photoacoustic tomography reconstruction based on in vivo mouse

**Fig. 8:**
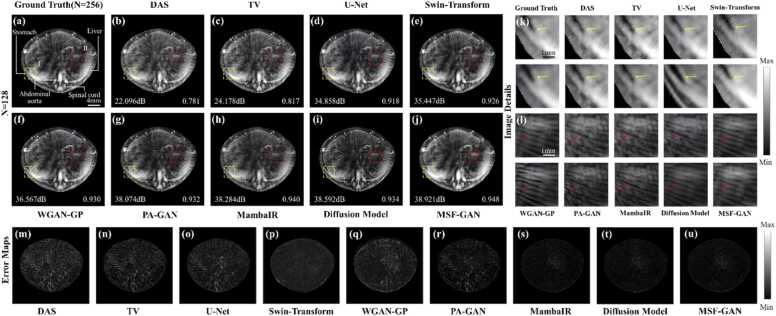
Reconstruction results of *in vivo* mouse under 128 views: (a) ground truth; (b) DAS; (c) TV; (d) U-Net; (e) Swin-Transformer; (f) WGAN-GP; (g) PA-GAN; (h) MambaIR; (i) Diffusion Model; (j) MSF-GAN; (k) and (l) magnified regions of interest corresponding to (a)-(j); (m)-(u) error maps corresponding to (b)-(j).

To further verify applicability in *in vivo* tissue, sparse-view photoacoustic experiments were conducted on the mouse trunk under 128 and 64 views. Figs. 8(a)-8(j) showed reconstruction results from the ground truth, DAS, TV, U-Net, Swin-Transformer, WGAN-GP, PA-GAN, MambaIR, diffusion model and MSF-GAN under 128 views. These results evaluated the performance of structure recovery and artifact suppression in a complex *in vivo* background. Overall, the TV, U-Net, Swin-Transformer, WGAN-GP, PA-GAN, MambaIR, diffusion model and MSF-GAN all reduced background artifacts to some extent and improved reconstruction quality. However, most of them showed over smoothing and incorrect texture enhancement, leading to inaccurate recovery of the liver contour and gastric details in Figs. 8(a)-8(i). To intuitively assess the differences among methods in restoring fine structures, the stomach and liver regions I and II were magnified in Figs. 8(k) and 8(l). The TV method still produced distorted stomach structures, due to excessive penalization of sparse sampling artifacts in Fig. 8(k). Other deep learning methods enhanced organ textures and suppress some stripe artifacts. But as indicated by arrows in Fig. 8(l), they produced incorrect organ enhancement and failed to recover accurate high frequency details. In contrast, MSF-GAN produced cleaner background and sharper organ structure at the global level in Fig. 8(j), by leveraging multiscale structural information in photoacoustic images. Particularly in abdominal region I and II, MSF-GAN showed continuous liver and stomach contours. It still preserved high frequency details of skin vessels, presenting texture features consistent with full-view sampling. To comprehensively evaluate the imaging performance, PSNR and SSIM were calculated based on the reconstruction results in Fig. 8. Under 128 views, MSF-GAN achieved SSIM of 0.948, significantly outperforming other methods. Meanwhile, PSNR improved to 38.921 dB, demonstrating superior suppression of stripe artifacts.

Figs. 8(m)-8(u) presented error maps comparing different reconstructions with the ground truth, to analyze reconstruction bias at organ and tissue interfaces. The error maps revealed that MSF-GAN achieved the smallest intensity difference from ground truth, indicating higher consistency in liver, stomach, and skin contours. Other methods exhibited varying degrees of residual errors within abdominal organs and boundaries, reflecting sensitivity to sparse sampling artifacts. These results demonstrated that MSF-GAN not only performed well in simulation experiments but also achieved high quality imaging *in vivo* PAT reconstruction.

**Fig. 9:**
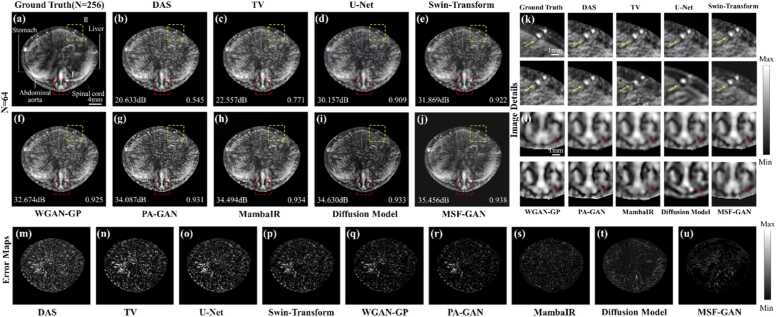
Reconstruction results of *in vivo* mouse under 64 views: (a) ground truth; (b) DAS; (c) TV; (d) U-Net; (e) Swin-Transformer; (f) WGAN-GP; (g) PA-GAN; (h) MambaIR, (i) Diffusion Model, (j) MSF-GAN; (k) and (l) magnified regions of interest corresponding to (a)-(j); (m)-(u) error maps corresponding to (b)-(j).

To further verify the reconstruction performance of MSF-GAN under extremely sparse conditions, mouse experiments were analyzed under 64 views. Figs. 9(a)-9(j) showed the ground truth and reconstruction results by DAS, TV, U-Net, Swin-Transformer, WGAN-GP, PA-GAN, MambaIR, diffusion model and MSF-GAN. Figs. 9(k) and 9(l) were the magnified regions, and Figs. 9(m)-9(u) showed the error maps.

From the overall and magnified regions, stripe artifacts obscured the original signals due to sampling view decreases, making abdominal organs almost indistinguishable in Fig. 9(b). TV method still cannot separate artifacts from true signals, leading to loss of structural information in region II in Fig. 9(c). U-Net and Swin-Transformer suppressed artifacts to some extent at low sampling rates, but the spine was clearly distorted in magnified region II in Figs. 9(d) and 9(e). WGAN-GP and PA-GAN outperformed the above methods in structural preservation and edge sharpness. But under extreme sparsity and complex backgrounds, some artifacts were mistakenly retained as textures, as marked by red arrows in Fig. 9(l). In addition, Figs. 9(h) and 9(i) showed that, both MambaIR and the diffusion model were able to recover part of the liver and stomach details under 64 views. However, overfitting was still observed in the region of interest. This was reflected by locally over-enhanced local skin texture structures and the generation of false details. These results suggest that, under extremely sparse sampling *in vivo* backgrounds, they still struggled to achieve a stable balance between faithful structural preservation and artifact suppression. In contrast, MSF-GAN maintained stable imaging performance even under very low sampling. The reconstructed images showed cleaner backgrounds and significantly reduced stripe artifacts in Fig. 9(j). Particularly at the yellow arrow in Fig. 9(k), blood vessels and skin structures were clearly visible. The spinal texture also showed more realistic morphology and closely resembled the ground truth in Fig. 9(l). PSNR and SSIM were also calculated based on the reconstruction results in Fig. 9. Under 64 views, MSF-GAN achieved the highest PSNR and SSIM values of 35.456 dB and 0.938. These advantages were attributed to the multiscale structural information and dual gradient adversarial constraints introduced in MSF-GAN. It enabled the model to capture features at multiple scales, while preserving high frequency details and suppressing artifacts under sparse conditions.

The error maps in Figs. 9(m)-9(u) further supported these findings. Other methods showed significant intensity variations, especially in visceral regions and skin details. In contrast, MSF-GAN reduced error magnitude overall, approaching the ground truth. The results demonstrated that MSF-GAN maintained high structural fidelity and texture consistency under extremely sparse sampling, confirming its superior reconstruction performance.

To comprehensively evaluate the imaging performance of different methods under various sparsity levels, PSNR and SSIM were calculated on the testing dataset. All quantitative results were reported as mean ± standard deviation over 50 images, as shown in Table 1. Although all methods improved PSNR and SSIM to varying degrees, their performance gains and stability differed noticeably. Among them, MSF-GAN achieved the best results under both sparse-view settings. Under 128 views, MSF-GAN achieved a PSNR of 38.919 ± 0.830 dB and an SSIM of 0.946 ± 0.011. Compared with the diffusion model and MambaIR, the PSNR and SSIM were improved by 0.434 dB and 0.011, corresponding to relative improvements of 1.128% and 1.176%, respectively. Under 64 views, MSF-GAN further demonstrated its superiority, achieving a PSNR of 35.450 ± 1.080 dB and an SSIM of 0.938 ± 0.015. PSNR and SSIM were 0.845 dB and 0.005 higher than those of the diffusion model and MambaIR, corresponding to relative gains of 2.442% and 0.536%, respectively. As shown in Table 1, when the number of sampling views decreased from 128 to 64, both PSNR and SSIM declined for all methods to different extents, indicating that increased sparsity substantially raised the reconstruction difficulty. Nevertheless, the proposed MSF-GAN maintained consistently superior performance across both under 128 views and 64 views. Moreover, MSF-GAN exhibited the smallest result fluctuations under both settings, further demonstrating that it not only achieved higher reconstruction accuracy but also provided better stability and robustness. These results confirmed that MSF-GAN maintained high fidelity structural recovery, providing a practical approach for high quality sparse-view PAT reconstruction.

**Table 1:** Quantitative comparison among different methods using evaluation metrics.

Sparse View	128		64	
Metrics	PSNR (dB)	SSIM	PSNR (dB)	SSIM
DAS	22.095 ± 1.862	0.779 ± 0.041	20.623 ± 2.746	0.540 ± 0.087
TV	24.169 ± 1.547	0.815 ± 0.033	22.550 ± 2.214	0.768 ± 0.052
U-Net	34.841 ± 1.213	0.917 ± 0.021	30.149 ± 1.684	0.904 ± 0.027
Swin-Transformer	35.434 ± 1.082	0.920 ± 0.018	31.858 ± 1.463	0.915 ± 0.023
WGAN-GP	36.552 ± 0.986	0.929 ± 0.016	32.654 ± 1.372	0.922 ± 0.021
PA-GAN	38.065 ± 0.911	0.931 ± 0.013	34.071 ± 1.240	0.930 ± 0.019
MambaIR	38.269 ± 0.882	0.935 ± 0.012	34.356 ± 1.147	0.933 ± 0.017
Diffusion Model	38.485 ± 0.866	0.932 ± 0.012	34.605 ± 1.101	0.932 ± 0.016
MSF-GAN	38.919 ± 0.830	0.946 ± 0.011	35.450 ± 1.080	0.938 ± 0.015

### 3.3 Ablation study

**Fig. 10:**
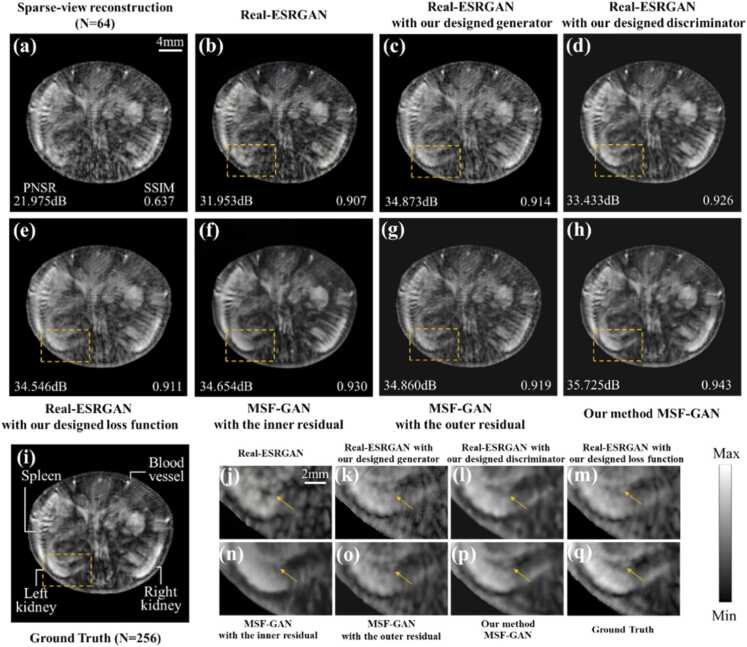
Ablation results of *in vivo* mouse under 64 views: (a) sparse view; (b) Real-ESRGAN; (c) Real-ESRGAN with our designed generator; (d) Real-ESRGAN with our designed discriminator; (e) ESRGAN with our designed loss function; (f) MSF-GAN without the inner residual; (g) MSF-GAN without the outer residual; (h) MSF-GAN; (i) ground truth; (j)-(q) magnified regions of interest corresponding to (b)-(i).

To evaluate the performance of different modules and loss functions, we conducted ablation studies using PAT *in vivo* reconstruction results under 64 views. We replaced corresponding components in Real-ESRGAN [46] with our proposed generator, discriminator, and loss function, to verify their effectiveness in artifact suppression and structure preservation. Figs. 10(a)-10(e) showed the sparse reconstruction image, Real-ESRGAN, Real-ESRGAN with our designed generator, Real-ESRGAN with our designed discriminator, Real-ESRGAN with our designed loss function, and MSF-GAN, respectively. To further justify the dual residual design in the generator, an ablation study was conducted by removing the inner residual connection in the PSA module and the outer residual connection in the Feature Enhance Block. Figs. 10(f)-10(h) presented MSF-GAN without the internal residual, MSF-GAN without the external residual and MSF-GAN. Fig. 10(i) presented the ground truth under 256 views. To further assess the enhancement effect of different modules, loss functions and residual connection on kidney structures, we analyzed the regions of interest marked by boxes in Figs. 10(b)-10(i).

The sparse reconstruction in Fig. 10(a) contained noticeable striping artifacts, which obscured parts of the left and right kidneys, leading to the loss of structural contours. Although Real-ESRGAN suppressed some artifacts and improved PSNR and SSIM, the boundaries still blended with background artifacts, particularly at the location indicated by the arrow in Fig. 10(j). In contrast, as shown in Figs. 10(c) and 10(k), Real-ESRGAN with our designed generator restored high frequency details of the kidneys. The improvement was attributed to the integration of the PSA module in the generator, which captured richer structural feature representation in photoacoustic imaging. However, in the overall image, artifacts in the central intestinal region were mistakenly enhanced, resulting in noticeable distortion. As shown in Fig. 10(d), Real-ESRGAN with our designed discriminator recovered the structures of the liver, kidneys, and intestines more accurately, though some reconstruction artifacts remained. The enhancement was achieved by skip connection with the CAM module in the discriminator, which provided both pixel and global level feedback, improving the structural consistency of organs. Figs. 10(e) and 10(m) demonstrated that Real-ESRGAN with our designed loss function better restored kidney contours. It was attributed to the dual gradient regularized adversarial loss integrated structural awareness from both the generator and discriminator. The qualitative and quantitative results in Figs. 10(f) and 10(g) demonstrated that removing either residual connection resulted in lower PSNR and SSIM values. As shown in Fig. 10(f), eliminating the internal residual connection caused blurring of fine vessel and kidney details, suggesting reduced ability to preserve local structural information. By contrast, removing the external residual connection in Fig. 10(g) degraded the overall structural continuity and introduced over enhanced regions, indicating impaired preservation of stable base features. These findings verified that the two residual connections served complementary functions and together contributed to improved sparse-view PAT reconstruction. In contrast, MSF-GAN accurately reconstructed abdominal organ structures in the *in vivo* mouse, combining the strengths of all three components. Particularly, as observed in Figs. 10(h) and 10(i) along with their corresponding regions of interest (Figs. 10(p) and 10(q)), the recovered kidney structures closely matched the ground truth. Quantitative results confirmed that MSF-GAN achieved the highest PSNR of 35.725 dB and SSIM of 0.943, indicating outstanding performance in superior artifact removal and structural preservation.

### 3.4 Three-dimensional sparse-view photoacoustic tomography reconstruction based on in vivo mouse

The mouse abdomen contains various organs and vessel structures, including the liver, spleen, kidneys and stomach. The abdominal environment is complex and layered, which exposes the ill-posed inversion issues in sparse-view photoacoustic tomography reconstruction. To verify the accuracy of MSF-GAN, three-dimensional *in vivo* PAT experiments were conducted on mouse trunk under 64 and 32 views. Representative 2D slices were analyzed to assess detail recovery and artifact suppression in complex biological tissue.

**Fig. 11:**
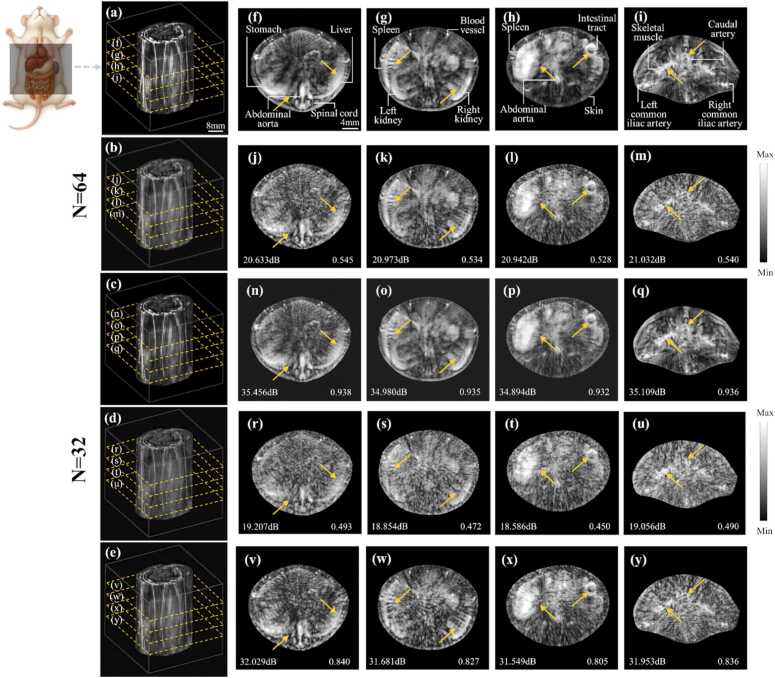
Three-dimensional results of mouse under 64 and 32 views: (a) 3D reconstruction results of ground truth; (b) 3D reconstruction results of DAS and 64 views; (c) 3D reconstruction results of MSF-GAN and 64 views; (d) 3D reconstruction results of DAS and 32 views; (e) 3D reconstruction results of MSF-GAN and 32 views; (f)-(y) results of upper abdomen, middle abdomen, lower abdomen and caudal trunk in (a)-(e).

Fig. 11(a) showed the three-dimensional PAT image of mouse trunk under full-view, where subcutaneous vessels were clear. Fig. 11(b) and Fig. 11(d) showed the DAS reconstruction results under 64 and 32 views. The vascular network of the mouse appeared discontinuous. Figs. 11(c) and 11(e) showed the three-dimensional sparse reconstruction result obtained by MSF-GAN, where the vessel network became continuous and clear. MSF-GAN achieved 75% and 88% improvement in imaging speed while maintaining imaging quality close to full sampling. It demonstrated that the method can preserve integrity and visibility of three-dimensional structures under extremely sparse views.

To further evaluate MSF-GAN performance across different tissue layers, we extracted four typical mouse trunk slices from the three-dimensional volumes in full views, 64 views, 32 views and MSF-GAN reconstruction. The four anatomical sections were shown in Figs. 11(f)-11(y), corresponding to upper abdomen, middle abdomen, lower abdomen, and caudal trunk. Comparing these regions allowed direct assessment of artifact suppression and structural fidelity in mouse trunk imaging. In the sparse-view result, Fig. 11(j) and (r) showed that the stomach and spinal cord in the upper abdomen were almost covered by artifacts. In Figs. 11(k), 11(l), 11(s) and 11(t), kidney and spleen boundaries in the middle and lower abdomen were blurred. In the kidneys, high frequency texture was missing and mixed, making them hard to distinguish. In contrast, the MSF-GAN reconstruction results in Figs. 11(n) and 11(v) showed the liver, stomach, aorta and spinal cord with clear boundaries in the upper abdomen, matching the full-view reconstruction results. In the middle and lower abdomen, MSF-GAN effectively suppressed background stripes and produced sharp structures in the kidneys and spleen, with clearly visible organ folds and textures in Figs. 11(o), 11(p), 11(w) and 11(x). In the caudal trunk, the fine striated muscle fibers were accurately recovered without over smoothing or false textures. Although the reconstruction quality at 32 views was lower than that at 64 views, MSF-GAN still preserved the overall anatomical organization of the mouse trunk. In the 32 views reconstruction, the major organs remained recognizable, including the liver, stomach, kidneys, spleen, aorta, and spinal cord. This trend was particularly evident in the kidneys and spleen Fig. 11(w), where some subtle internal details became less distinct under more severe under sampling. Nevertheless, the principal organ contours and relative distributions were still maintained, indicating that MSF-GAN retained structural reconstruction ability even under extremely sparse view conditions. Across the four representative slices, MSF-GAN consistently outperformed DAS at both 64 and 32 views. At 64 views, the average PSNR increased from 20.895 dB to 35.110 dB, while the average SSIM increased from 0.537 to 0.935. At 32 views, the average PSNR increased from 18.926 dB to 31.803 dB, and the average SSIM increased from 0.476 to 0.827. These results further confirmed the MSF-GAN can robustly restore the complex distribution of organs and skin vessels in the mouse trunk.

The overall experiment results confirmed that MSF-GAN accurately reconstructed organ contours and vascular textures of the mouse trunk under sparse sampling. By combining multiscale structural information, MSF-GAN effectively extracted hierarchical features from photoacoustic signals. Combined with the dual-gradient adversarial loss, it stably suppressed ill-posed inversion artifacts caused by sparse-view reconstruction. It reduced sampling requirements while preserving true structural characteristics of biological tissues. MSF-GAN provided a practical solution for high speed and quality reconstruction in *in vivo* animals, demonstrating broad potential in biomedical applications.

## 4 Discussion

Sparse sampling has gradually become an effective technique to balance imaging speed and quality. However, limited views can cause inverse problems such as artifacts and loss of details, which severely degrade image quality. Therefore, developing a PAT reconstruction method that can maintain structural fidelity while suppressing artifacts under sparse sampling is of great practical importance. To address this issue, this paper develops a sparse-view photoacoustic tomography system and proposes a generative adversarial network that incorporates multiscale structural features for high quality reconstruction. By introducing a pyramid squeeze attention block and a skip connection with channel attention module, MSF-GAN enhances the ability to recover fine structural details from sparse-view data. And a dual-gradient regularized adversarial loss function is designed to improve the stability of artifact suppression. Experimental results demonstrate that under sparse-view conditions, MSF-GAN reconstructs high quality organ structures and maintains strong contrast in complex biological backgrounds. It significantly reduces photoacoustic data volume and preserves structural textural features comparable to full-view sampling, showing advantages in sparse-view PAT reconstruction. While other deep learning methods often suffer from high data dependency and spurious structural details under sparse-view conditions, MSF-GAN overcomes these limitations and achieves more accurate reconstruction. MSF-GAN effectively addresses the challenges arising from the rapid increase in acquisition channels and data volume with the advancement of three-dimensional and wide field PAT systems.

Although MSF-GAN provides a solution for high quality PAT sparse reconstruction, the method remains improvable in several respects. The training and evaluation of MSF-GAN are mainly based on the internally developed photoacoustic tomography system. Applying the model to different imaging systems may introduce distribution shifts in the measured signals, thereby limiting its performance of cross domain. The issue is more pronounced in sparse-view photoacoustic tomography. For example, variations in transducer response or sparse sampling patterns may result in different levels of streak artifacts and structural distortion. To address the issue, future work will focus on improving the generalization ability of MSF-GAN under imaging conditions from multiple systems. A possible solution is to adopt a joint training strategy by constructing datasets that include multiple photoacoustic imaging systems and acquisition settings. It allows MSF-GAN to be trained on more diverse sparse-view photoacoustic data. In addition, introducing diverse parameter variations during training, such as changes in sparse sampling angles and transducer characteristics, can improve its adaptability across different photoacoustic imaging conditions. The domain adaptation strategies also can be introduced to reduce the distribution discrepancies between different systems. MSF-GAN is expected to enforce consistency in the feature space across different photoacoustic systems and sparse acquisition settings, thereby more effectively suppressing artifacts caused by sparse sampling. The current study mainly focuses on single modality photoacoustic imaging, while multimodal imaging represents an important direction for future development. Combining photoacoustic with ultrasound or elastography could enable a more comprehensive visualization of internal biological structures. Integrating MSF-GAN into multimodal frameworks could provide accurate tissue information and higher imaging speed for real time monitoring, offering reliable evidence for disease diagnosis and treatment. In addition, the image reconstruction was performed using a homogeneous acoustic model, assuming a constant sound speed of 1500 m/s. The simplification improves computational efficiency and ensures consistency across different reconstruction methods. However, it should be noted that acoustic heterogeneity in biological tissues may degrade reconstruction quality, such as causing phase mismatches and localization errors. Therefore, we will future focus on incorporating heterogeneous acoustic models. First, dual speed or multicompartment modeling can be employed, in which different sound speeds are assigned to different propagation regions to provide a more realistic approximation of acoustic wave propagation in biological tissues. Second, ultrasound guided heterogeneous sound speed correction also represents a promising direction, as co-registered ultrasound information can be exploited to estimate spatially varying sound speed distributions and integrate them into the reconstruction procedure for improved accuracy. Furthermore, the incorporation of sound speed distribution information into a generative adversarial framework could improve reconstruction accuracy in complex *in vivo* environments. Moreover, the current system uses a single wavelength for photoacoustic excitation. Applying multi-wavelength laser excitation and contrast agent could achieve functional imaging and molecular imaging. Under these application conditions, the quantitative performance of the reconstruction model becomes a critical issue, which requires the network preserves quantitative photoacoustic amplitudes both spatially and spectrally. The proposed MSF-GAN trained with gradient based loss, is expected to maintain the relative amplitude relationships in the reconstructed images. In future work, additional strategies, such as spectral consistency, will be integrated into the MSF-GAN framework. The enhanced MSF-GAN could enhance the speed and quantitative stability of functional imaging while maintaining structural accuracy, providing additional information dimensions for disease diagnosis. Overall, the proposed MSF-GAN achieves a balance between imaging quality and high speed performance. Future work will extend its application to multimodal and functional imaging, thereby improving the value of photoacoustic imaging for physiological monitoring and precise disease diagnosis, and expanding its potential in clinical and biomedical research.

## 5 Conclusion

In this paper, we have developed a sparse-view photoacoustic tomography system and generative adversarial network with multi-scale structural feature for real time photoacoustic reconstruction. The proposed PAT system effectively reduces data volume, while improving the quality of sparse reconstruction. MSF-GAN incorporates pyramid squeeze attention module in the generator to extract multi-scale structural features of photoacoustic image, and introduces skip connections with channel attention modules in the discriminator to enhance structural consistency. The dual-gradient regularized adversarial loss is designed to stabilize training and balance artifact suppression with fine detail enhancement. The simulation and *in vivo* experimental results verify that MSF-GAN not only restores clear organ structures but also maintains high contrast under complex backgrounds. Quantitative analysis of 64 views sparse *in vivo* reconstruction indicates that MSF-GAN achieves the highest PSNR and SSIM values of 35.450 ± 1.080 dB and 0.938 ± 0.015. It confirms that MSF-GAN achieves image quality close to full-view imaging under extremely limited data conditions. The work can be further applied to biomedical applications such as diagnosis and treatment in *in vivo* animals, offering technical support for the photoacoustic clinical translation.

## CRediT authorship contribution statement

**Yi Shen:** Supervision, Funding acquisition. **Zheng Yuan:** Investigation. **Tingting Li:** Writing – original draft, Validation. **Gang Li:** Data curation. **Yiming Ma:** Supervision. **Xudong Luo:** Data curation. **Jialin Li:** Writing – review & editing, Writing – original draft, Visualization, Validation, Software, Resources, Methodology, Investigation, Formal analysis, Data curation, Conceptualization. **Mingjian Sun:** Writing – review & editing, Supervision, Resources, Project administration, Funding acquisition, Conceptualization. **Cheng Ma:** Writing – review & editing, Supervision, Investigation, Conceptualization.

## Declaration of Competing Interest

The authors declare that they have no known competing financial interests or personal relationships that could have appeared to influence the work reported in this paper.

## Data Availability

Data will be made available on request.
