# Supplementary material for: A generative adversarial network with multi-scale structural features for sparse-view photoacoustic tomography reconstruction
Source: Photoacoustics. 2026 Apr 28;49:100828. doi: 10.1016/j.pacs.2026.100828 (PMC13142112; doi:10.1016/j.pacs.2026.100828)
Supplement: Supplementary file 1 — Supplementary material [file mmc1.docx]

supplement material

A generative adversarial network with multi-scale structural features for sparse-view photoacoustic tomography reconstruction


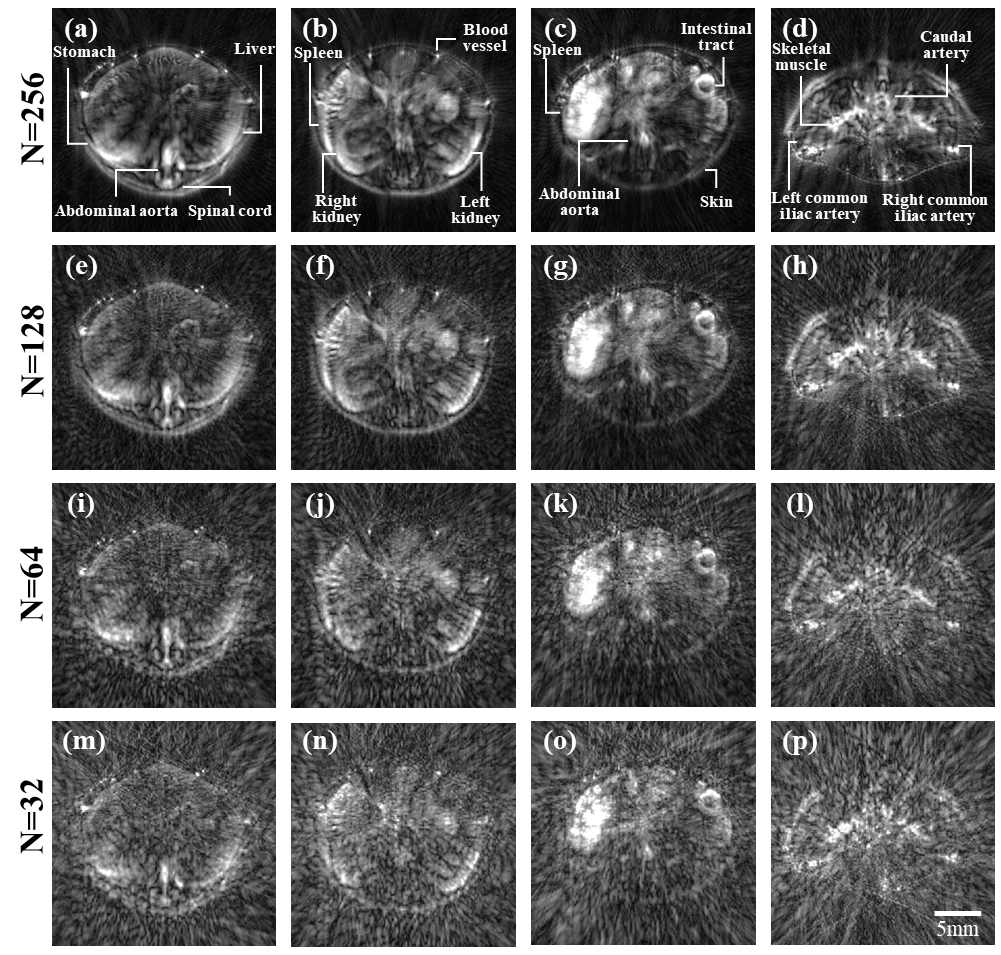


Fig. S1. Representative DAS reconstruction results of the *in vivo* dataset without post-processing: (a)-(d) ground truth; (e)-(h) 128 views; (i)-(l) 64 views; (m)-(p) 32 views.


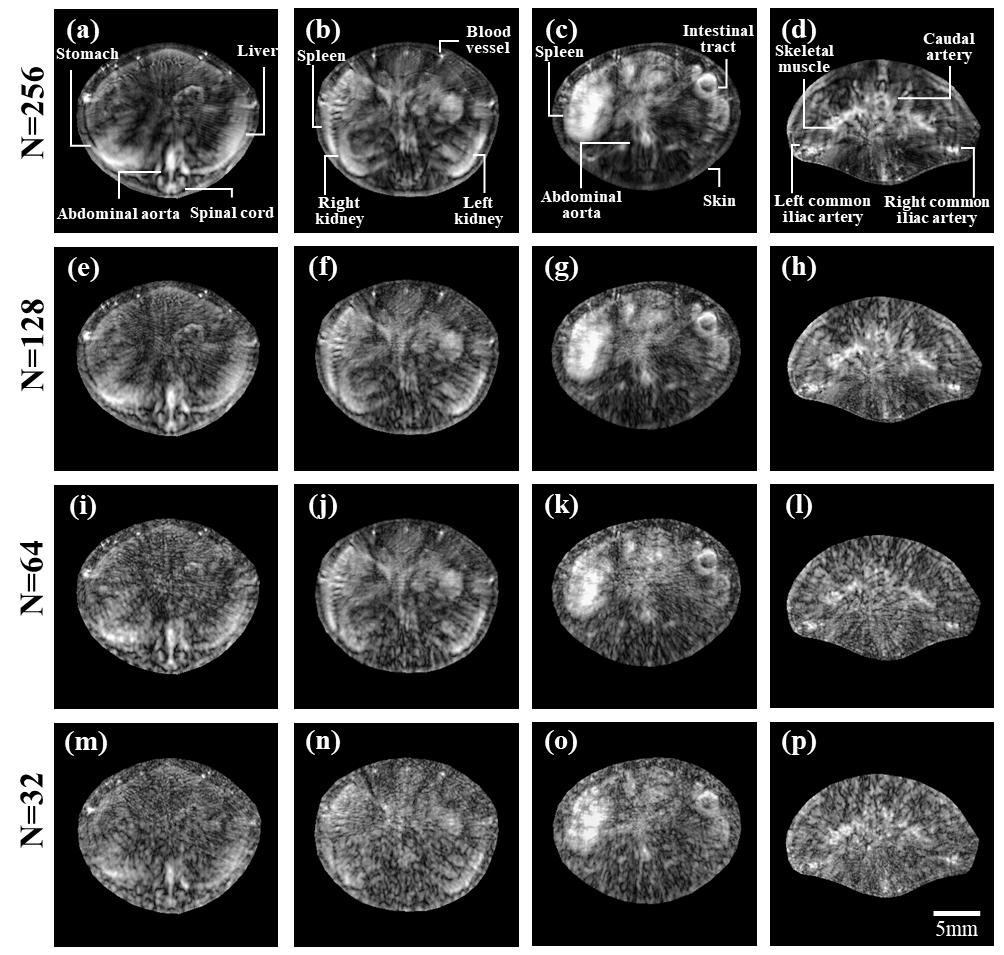


Fig. S2. Representative DAS reconstruction results of the *in vivo* dataset after post-processing: (a)-(d) ground truth; (e)-(h) 128 views; (i)-(l) 64 views; (m)-(p) 32 views.
